# Functional Connectivity’s Degenerate View of Brain Computation

**DOI:** 10.1371/journal.pcbi.1005031

**Published:** 2016-10-13

**Authors:** Guillaume Marrelec, Arnaud Messé, Alain Giron, David Rudrauf

**Affiliations:** 1 Sorbonne Universités, UPMC Univ Paris 06, CNRS, INSERM, Laboratoire d’imagerie biomédicale (LIB), Paris, France; 2 Department of Computational Neuroscience, University Medical Center Eppendorf, Hamburg University, Hamburg, Germany; 3 Grenoble Institute of Neuroscience, INSERM-UJF-CHU, Grenoble, France; Ghent University, BELGIUM

## Abstract

Brain computation relies on effective interactions between ensembles of neurons. In neuroimaging, measures of functional connectivity (FC) aim at statistically quantifying such interactions, often to study normal or pathological cognition. Their capacity to reflect a meaningful variety of patterns as expected from neural computation in relation to cognitive processes remains debated. The relative weights of time-varying local neurophysiological dynamics versus static structural connectivity (SC) in the generation of FC as measured remains unsettled. Empirical evidence features mixed results: from little to significant FC variability and correlation with cognitive functions, within and between participants. We used a unified approach combining multivariate analysis, bootstrap and computational modeling to characterize the potential variety of patterns of FC and SC both qualitatively and quantitatively. Empirical data and simulations from generative models with different dynamical behaviors demonstrated, largely irrespective of FC metrics, that a linear subspace with dimension one or two could explain much of the variability across patterns of FC. On the contrary, the variability across BOLD time-courses could not be reduced to such a small subspace. FC appeared to strongly reflect SC and to be partly governed by a Gaussian process. The main differences between simulated and empirical data related to limitations of DWI-based SC estimation (and SC itself could then be estimated from FC). Above and beyond the limited dynamical range of the BOLD signal itself, measures of FC may offer a degenerate representation of brain interactions, with limited access to the underlying complexity. They feature an invariant common core, reflecting the channel capacity of the network as conditioned by SC, with a limited, though perhaps meaningful residual variability.

## Author Summary

The human brain is characterized by both the way its neurons are connected (the anatomy) and the way they emit signals to interact (the dynamics). At the typical scale of measurements, by analogy with the road network, anatomy (the ‘roads’) is expected to remain relatively stable over time and reproducible from person to person, while neuronal dynamics propagating in the network (the ‘traffic’) can be expected to vary widely, both over time and between people. But, paradoxically, patterns of functional connectivity (FC), a convenient proxy to quantify interactions between pairs of brain regions in magnetic resonance imaging (MRI), have been found to be reproducible both within and between subjects across several studies, while other studies, on the contrary, have featured variable FC patterns, often in relation to cognitive processes. We investigate this unsettled issue quantitatively using multivariate statistics and generative models of brain activity. We show, across a range of estimators, that FC offers a degenerate representation of brain interactions and strongly depends on anatomy.

## Introduction

The processing and routing of information in the brain are implemented through the interplay of two general constraints: 1) the specifics of physiological dynamics, controlling how connected neurons respond to each other locally, and 2) the entire wiring diagram of anatomical connections, channeling the possible exchange of information between neuronal ensembles according to the properties of its cables [1–6]. While large-scale anatomical connections are expected to remain relatively stable over time, neuronal processes may be expected to produce complex transient patterns related to different processing phases, either in a stationary or non-stationary manner [7]. Since the same anatomical network may be used to implement variable physiological dynamics at different times [5, 8, 9], many degrees of freedom could be expected in the relationship between structure and function, that is, between the wiring diagram and the resulting patterns of activity [5, 10–12].

In the context of neuroimaging, the wiring diagram of connections, or ‘structural connectivity’ (SC), has generally been estimated using DWI-based tractography [13–15]. In blood oxygen level dependent (BOLD) resting-state functional magnetic resonance imaging (rs-fMRI, where subjects passively lie in the magnet [16, 17]), measures of ‘functional connectivity’ (FC) have emerged as a convenient proxy to quantify functional patterns of interaction between neural ensembles [16, 18–21]. In this scientific field, standard Pearson correlation has become a gold standard [22–24]; more elaborate measures, though rarely applied so far, have also been proposed, such as mutual information as well as estimators of higher-order relationships [25–30]. If FC were sensitive to features of dynamics related to ongoing cognition, we could expect a relatively large variability and complexity of this measure, in particular as compared to SC. In the resting state, for instance, cognition is allowed to roam free range, and FC could thus be expected to be quite variable across arbitrary acquisition times, both within and between subjects. Beyond theoretical rationale, empirical evidence displays mixed results making FC hard to assess as a measure. Several studies support the hypothesis that FC can express a variety of patterns and complex dynamical behaviors, which can demonstrate empirical correlations with normal and pathological cognitive processes [31]. As a matter of fact, it has already been shown that FC could be sensitive to brain maturity [32], age [33–38], the global level of awareness [8, 39], personality traits [40, 41], the current mental state [31, 42, 43], recent experience [44], and varies over time for a given subject [8, 39]. It has even been suggested that the between-subject differences could serve as individual fingerprints [45, 46]. Likewise, the variability of FC patterns across time, as observed with sliding time windows shorter than a standard acquisition run (‘dynamic FC’) has been recently featured with relation to cognition [47]. Yet, the origins of such differences and variations, whether structural, neuronal, hemodynamic/metabolic, or methodological, and their relative weights in the generation of FC—and thus the potential of FC as a measure—remain to be clarified [38, 48–51]. Besides, many other studies have suggested that FC reflects SC [22–24, 52–54] and patterns of FC extracted from rs-fMRI have been repeatedly found to be reproducible both within and across subjects [55–57]. Even though robust FC patterns can be related to general task-based networks from activation studies [58–64], overall these results suggested that the sensitivity of both the BOLD signal and the different measures of FC to the variability and complexity of the underlying dynamics might be quite limited [65]. The fact that FC is related to SC (which is relatively reproducible across subjects and stable over time—at least as seen by DWI tractography [66]), suggests a FC that would itself reflect the wiring diagram expressed in SC. Evidence for FC patterns that are both reproducible across subjects and sensitive to various subject-specific factors suggests that FC as usually measured might be composed of an invariant core that accounts for a large part of the variance, and a residual variable part. FC might thus have existing but limited sensitivity to important features of brain dynamics, while being strongly dependent on more invariant factors of interaction such as SC, its communication channel capacity and global steady routing schemes. FC would therefore appear as a degenerate measure of the expected complexity of brain interactions, all the more so that it would reflect SC, which, as a measure of static anatomical wiring diagram, implies the highest degeneracy with respect to brain dynamics [11]. Likewise, it remains unclear how this could depend on the kind of measure used to quantify FC or the time window considered. Addressing these questions is important as they concern the potential of FC for capturing relevant aspects of the complexity of brain dynamics and our ability to infer meaningful neurocomputational properties from such measures.

**Fig 1:**
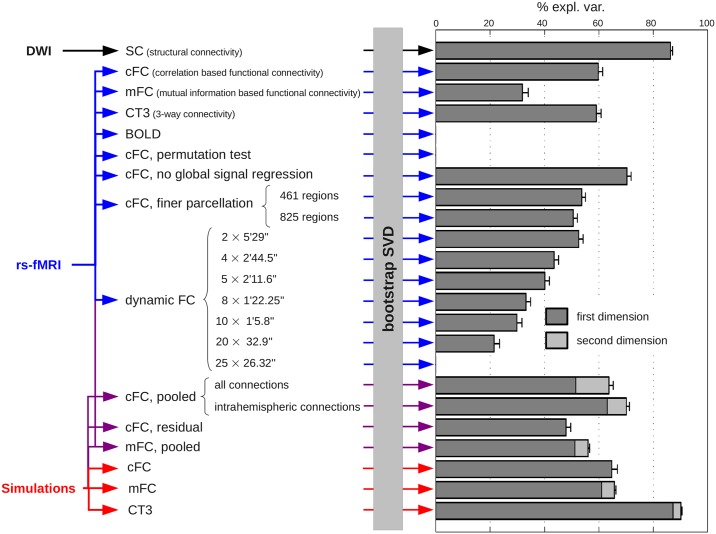
Summary of analyses. We performed bootstrap SVD of various measures of structural and functional connectivity. In each case, we represented the dimension of the reproducible linear subspace extracted by bootstrap SVD, the part of variance explained by each dimension (bootstrap average) as well as the (bootstrap) standard deviation associated to all reproducible dimensions.

In the present study, we characterized and compared qualitatively and quantitatively the relative variability of patterns of SC and FC, across different measures of FC (correlation, mutual information, 3-way connectivity, dynamic FC). We assessed: 1) the relative sensitivity of FC to variable states and dynamics as putatively underlying different phases and modes of brain activity, and 2) the extent to which FC reflects SC. We used DWI-based estimates of SC and rs-fMRI based estimates of FC from 21 normal participants over a partition of the brain into 160 regions [54]. In addition to the analysis of empirical data, in order to systematically assess the robustness of FC to different types of dynamics, we performed simulations of FC using seven different mainstream computational models of neuronal activity and BOLD response, with a wide range of complexity [54, 67, 68]. We used a unified analysis framework based on singular value decomposition (SVD) of matrices of connectivity and bootstrap statistics in order to compare patterns of connectivity between and within subjects and assess their variability in a multivariate framework. For each connectivity pattern under investigation, this method extracted a reproducible linear subspace with limited dimension that had the key feature of explaining the most variance or, equivalently, best explaining the data in a least square sense for that dimension (see Fig 1 and Methods).

## Results

### Empirical SC and FC mostly vary along one main linear dimension and are related

#### Empirical connectivity

**Fig 2:**
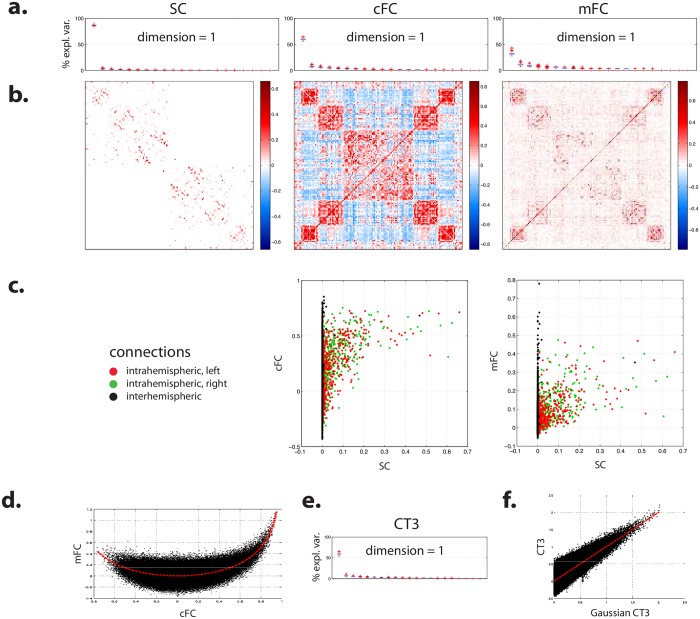
Empirical SC and FC. (**a**) Boxplot of the part of variance explained by the successive linear dimensions obtained by bootstrap SVD for SC (left), correlation based FC (cFC, middle), and mutual information based FC (mFC, right). (**b**) Average projection of data onto first dimension for SC (left), cFC (middle), and mFC (right). (**c**) Relationship between SC and FC according to these average projections for cFC (left) and mFC (right). (**d**) Relationship between empirical cFC and mFC. The dotted line is the relationship that holds in the case of a bivariate normal distribution, see Eq (1). (**e**) Boxplot of the fraction of variance explained by the successive dimensions obtained by bootstrap SVD of 3-way connectivity (CT3). (**f**) Relationship between empirical CT3 and CT3 computed under the assumption of a trivariate Gaussian distribution, see Eq (2). The dotted line stands for the identity, that is CT3 = Gaussian CT3. See §3 of S1 Text for a view of the reproducible part of empirical SC, cFC, and mFC projected on anatomical template.

We first characterized the variability of the patterns of SC, correlation based FC (cFC), and mutual information based FC (mFC) across subjects. For all three measures, bootstrap SVD extracted a reproducible linear subspace with dimension 1 and 86.3% ± 0.8% for SC, 59.8% ± 1.6% for cFC and 31.8% ± 2.2% for mFC of variance explained (Fig 2a). All measures were essentially organized along one linear dimension and differences between individual connectivity patterns basically reduced to a global multiplicative constant. Thus about 86% of the variance in SC was either due to similarity in structure among subjects or in imaging biases; only about 14% of the variance could be accounted for by individual variability or imaging noise, and did so in a manner that did not indicate any particular linear direction. Similar statements hold for cFC and mFC, even though the lower fraction of variance explained for mutual information suggested that the corresponding patterns of connectivity could not be reduced as much to one linear dimension.

#### Relationships between SC and FC

The patterns of both empirical SC and FC reproducibly projected onto a one-dimensional linear subspace, reflecting in both cases an invariant common core only varying along a global multiplicative constant (Fig 2b). Furthermore, we found a relationship between these structural and functional cores (Fig 2c). However, the remaining unexplained variance, which was distributed across all the other dimensions with no reproducible linear direction, was smallest for SC (14%), intermediate for cFC (40%) and largest for mFC (68%).

#### Relationships between cFC and mFC

Empirical correlation and mutual information appeared to be roughly related, with a broad residual variance, through

$$\text{mFC}=-\frac{1}{2}\ln\left(1-{\text{cFC}}^{2}\right),$$ (1)

which exists between correlation and mutual information when the underlying data are generated according to a bivariate Gaussian process (Fig 2d).

#### 3-way connectivity

We reasoned that bivariate measures such as correlation and mutual information could potentially overlook more complex interaction patterns between regions, in particular those involving three regions or more, and that such patterns might not be as much reducible to a one-dimensional linear subspace as those derived from analyses constrained by a bivariate framework. We thus computed 3-way connectivity (CT3) for each triplet of regions (see Methods). Application of the bootstrap SVD framework to this measure extracted a reproducible linear subspace with dimension 1 and explaining 59.0% ± 1.8% of variance (Fig 2e). The relationship between cFC and CT3 was strong and again suggested the existence of an underlying Gaussian process (Fig 2f).

#### The degeneracy of FC is a consequence of the anatomical organization of regions and links

To confirm that the degeneracy consistently observed was not due to a bias of the method but a consequence of the anatomical organization of regions and links, we performed three permutation tests. In the first permutation test, we assumed that the labeling of the links had no influence on the degeneracy observed; for each subject, we therefore randomly permuted the labels corresponding to the 12 720 links. In the second permutation test, we assumed that it is the labeling of the regions that had no influence on the degeneracy observed; for each subject, we therefore randomly permuted the labels corresponding to the 160 regions. In the third and last permutation test, we assumed that the labeling of regions within one hemisphere had no influence on the degeneracy observed; for each subject, we therefore randomly permuted the labels of the 80 regions within one hemisphere, while keeping the pairing between homologous regions. In none of the three cases did the analysis extract a reproducible linear subspace. Variance was not concentrated on the first linear dimensions, and the first three dimensions only accounted for a limited fraction of the variance (16.6% ± 3.9%, 12.7 ± 2.0%, 10.8% ± 1.5% for the first test; 16.5% ± 3.7%, 12.7 ± 2.1%, 10.9% ± 1.5% for the second test; 17.5% ± 3.4%, 13.0 ± 1.8%, 10.9% ± 1.3% for the last test). These results show that the anatomical organization of regions and links matters in the degeneracy observed.

#### The degeneracy of FC is not merely a translation of the degeneracy of the BOLD signal itself

Bootstrap SVD of both cFC and mFC yielded a one-dimensional reproducible linear subspace, which explained a substantial amount of the total variance. We wondered whether this could rather reflect a limiting degeneracy of the BOLD signal itself with respect to the complexity of the underlying brain computation, as opposed to a relative degeneracy introduced by the act of measuring FC. In order to approach this issue, we performed a bootstrap SVD analysis of the BOLD signal time series themselves (see Methods). The analysis did not extract any reproducible linear subspace. Variance was not concentrated on the first linear dimensions: the first three dimensions only accounted for 19.2% ± 5.1%, 13.6% ± 2.8%, and 10.9% ± 1.8% of the variance respectively. Altogether, these results indicate that BOLD data cannot be reduced at all to a linear space of low dimension. Either the data are of full dimension, or the relationship that exists between them cannot efficiently be approximated by a linear relationship.

#### The relative degeneracy of FC is not likely to be a consequence of the preprocessing step

To assess whether the degeneracy of FC could be a consequence of the preprocessing of fMRI data, we modified it in two directions. First, we considered two alternative parcellation schemes at finer scales (461 and 825 regions instead of 160, see Methods). We also computed cFC from the data without regression of the global signal. In the three cases, bootstrap SVD extracted a reproducible linear space with dimension 1 explaining 53.7% ± 1.4% (for a 461 regions), 50.5% ± 1.5% (for 825 regions), and 70.3% ± 1.5% (no global signal regression) of variance.

### Simulated FC from models with different dynamical behaviors are similar and strongly related to SC

We then adopted an approach based on computational modeling as a heuristic tool to study the sensitivity of FC to the specificity of the dynamical behaviors controlling interactions between connected regions. We generated simulated FC based on an array of standard computational models, all taking SC as input but with broadly different dynamical behaviors and equations. We wondered whether the patterns of FC generated by the different models would be able to fit empirical FC in the same manner or would retain irreducible components proper to the specific properties of the models (see Methods).

#### Simulated FC

**Fig 3:**
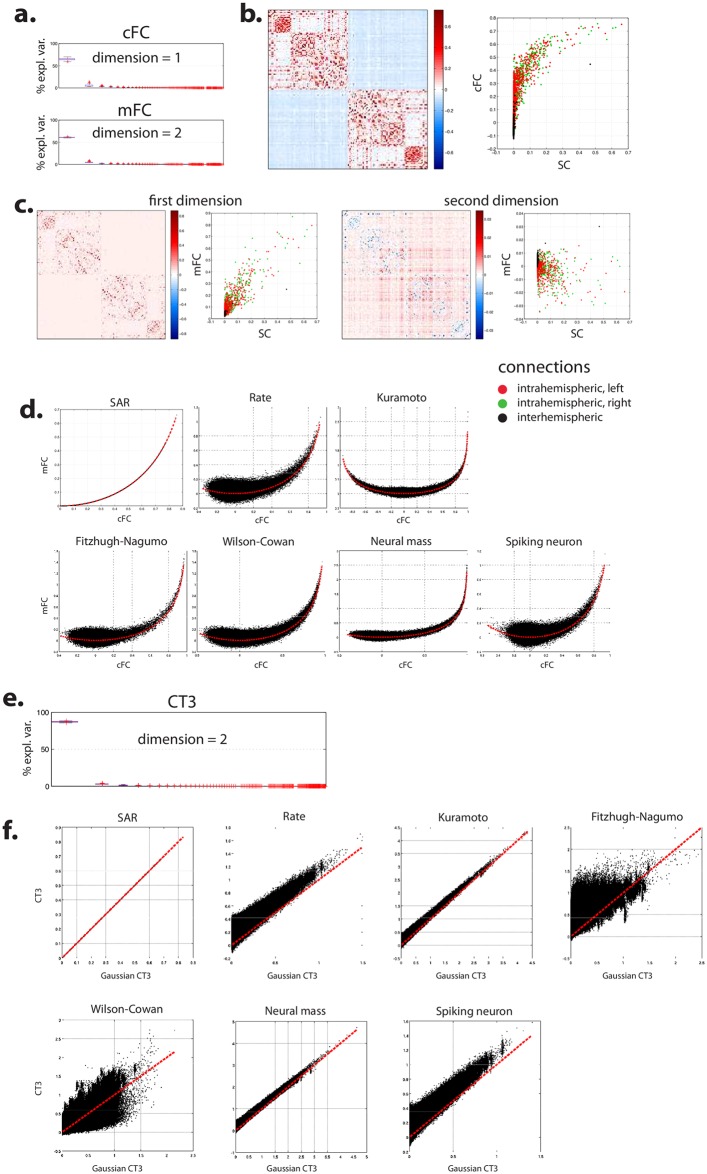
Robustness of simulated FC to dynamics. (**a**) Boxplot of the part of variance explained by the successive linear dimension obtained by bootstrap SVD for cFC (top) and mFC (bottom). (**b**) Average projection of data onto first dimension of cFC: representation (left) and relationship as a function of the average projection of empirical SC onto its first dimension (right). (**c**) Average projection of data onto first and second dimensions of mFC: representation and relationship as a function of the average projection of empirical SC onto its first dimension. **(d)** Relationship between simulated cFC and mFC for the different generative models used. The dotted lines stand for the relationship that hold for a bivariate Gaussian distribution, see Eq (1). (**e**) Boxplot of the part of variance explained by the successive linear dimensions obtained by bootstrap SVD for simulated CT3. (**f**) Relationship between empirical CT3 and CT3 computed under the assumption of a trivariate Gaussian distribution for the different generative models used, see Eq (2). The dotted line stands for the identity, that is CT3 = Gaussian CT3. See §3 of S1 Text for a view of the reproducible part of simulated cFC and mFC projected on anatomical template.

Across models and subjects, bootstrap SVD of simulated FC yielded a reproducible linear subspace with dimension 1 for cFC (64.7% ± 2.1% of explained variance) and 2 for mFC (61.0% ± 0.5% and 4.7% ± 0.8% of explained variance) (Fig 3a). Thus simulated FC appeared quite robust to basic differences in dynamical constraints.

#### Relationships with SC

Similar to empirical FC, simulated FC reproducibly projected onto a restricted one- or two-dimensional linear subspace. The variance unexplained by the first dimension was limited and this time similar for cFC (35%) and mFC (39%), though mFC induced a second reproducible dimension of small explanatory variance that was not observed with cFC. The first dimension of FC appeared related to that of SC for both cFC and mFC (Fig 3b and 3c): the stronger the values of SC, the stronger the values of FC; however, the pattern of mFC along the second reproducible dimension did not appear related to SC (Fig 3c).

#### Relationship between cFC and mFC

Overall, cFC and mFC appeared to be strongly related. The overall shape of the relation fitted again the analytical relation described by Eq (1) (Fig 3d). Contrary to the results obtained with empirical FC, the residual variance from the Gaussian model was small with simulated data for most computational models.

#### CT3

As for empirical data, we applied the bootstrap SVD framework to simulated CT3. The analysis yielded a reproducible linear subspace with dimension 2 (87.3% ± 0.5% and 2.9% ± 0.2% of variance explained) (Fig 3e). The relationship between simulated cFC and simulated CT3 was strong and suggested the existence of an underlying Gaussian process (Fig 3f).

### Relationship between simulated and empirical FC is strong, limited by errors in DWI-based estimate of SC, and driven by SC

**Fig 4:**
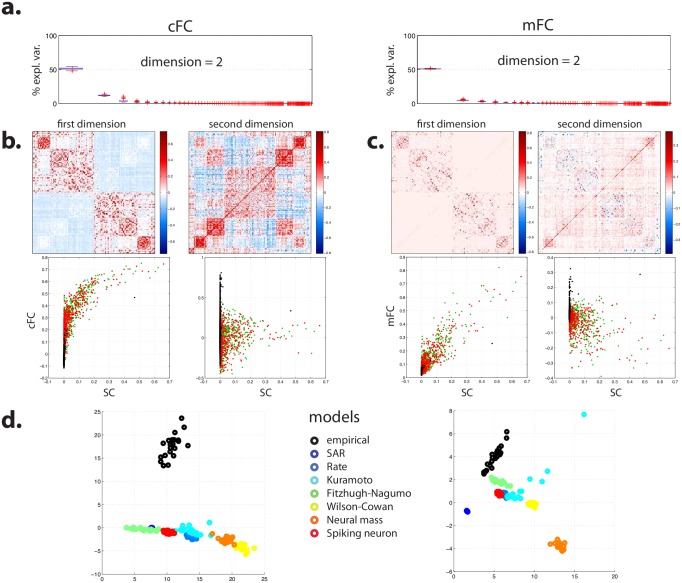
Joint SVD of simulated and empirical FC. (**a**) Boxplot of the part of variance explained by the successive linear dimensions obtained by bootstrap SVD for cFC (left) and mFC (right). (**b**) Average projection of data onto first (left) and second (right) dimensions for cFC: representation (top) and characterization as a function of the average projection of empirical SC onto its first dimension (bottom). **(c)** Same information for mFC. **(d)** Loading of data on the first and second dimensions broken down by model and empirical data, for cFC (left) and mFC (right). See §3 of S1 Text for a view of the reproducible part of cFC and mFC projected on anatomical template for pooled data.

We then assessed whether pooled empirical and simulated FC across subjects would project onto the same small number of dimensions by performing a global bootstrap SVD analysis. We found a reproducible linear subspace with dimension 2 (63.7% ± 1.6% of variance explained for cFC and 56.0% ± 0.5% for mFC) (Fig 4a). In both cases, the first dimension was tightly related to intrahemispheric connections, while the second dimension largely reflected homotopic and, more largely, interhemispheric connections (Fig 4b and 4c). For both cFC and mFC, the first dimension appeared again tightly related to SC, but not the second one. Finally, it was mostly empirical FC (which, contrary to simulations, demonstrated clear patterns of interhemispheric connections) that loaded onto the second dimension (Fig 4d).

We reasoned that customary errors of DWI-based SC estimation, concerning more particularly homotopic and interhemispheric connections, could account for the appearance of a second linear dimension when adding empirical FC to simulated FC. Homotopic and interhemispheric connections are known to exist but to be difficult to measure, remaining largely undetected by standard DWI acquisition and processing pipelines [54, 69, 70]. Such connections were mostly absent of the SC dataset that we used as an input to the generative models.

**Fig 5:**
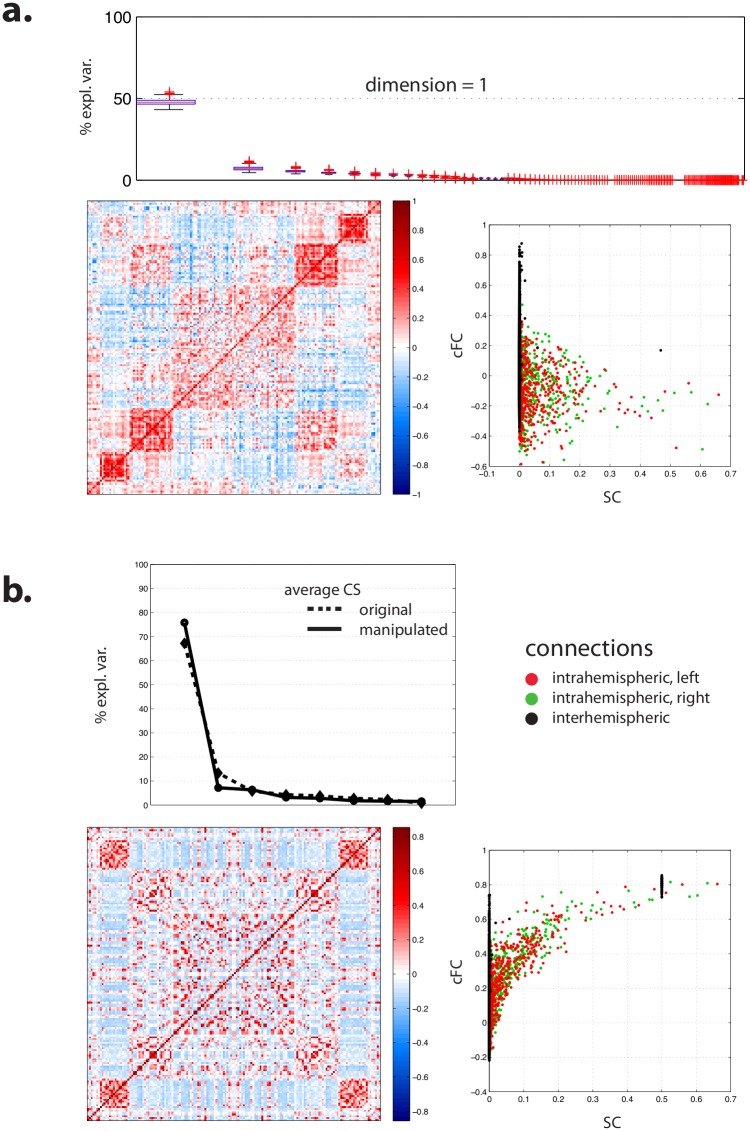
Joint SVD of simulated and empirical FC (continued). (**a**) Bootstrap SVD for residual cFC, computed as the difference between empirical and simulated cFC: boxplot of the part of variance explained by the successive linear dimensions (top), average projection of data onto first dimension (bottom left) and relationship of this projection as a function of the average projection of empirical SC onto its first dimension (bottom right). (**b**) SVD of data from average subject, where empirical SC and FC are computed by averaging across subjects. Simulated FC is obtained by feeding the average SC to the different generative models, either in its original form or after artificially setting all homotopic connections to an arbitrary value. Top: part of variance explained by the successive linear dimensions; bottom left: average projection of manipulated data onto first dimension; bottom right: relationship of this projection with manipulated average SC.

First, we investigated residual cFC by computing the difference between empirical and simulated FC for each generative model and subject. A bootstrap SVD analysis yielded a reproducible linear subspace with dimension 1 (47.9% ± 1.7% of explained variance). This dimension saliently featured interhemispheric and, more specifically, homotopic connections (Fig 5a). Thus a substantial amount of the differences between empirical and simulated FC could be reduced to errors pertaining to homotopic connections.

Second, we performed an SVD analysis in which simulated and empirical cFC were pooled together but on intrahemispheric connections only, as a way of excluding putative errors of prediction directly and explicitly related to interhemispheric connections. This analysis yielded a reproducible linear subspace with dimension 2. Nonetheless, compared to bootstrap SVD over all (intra- and interhemispheric) connections, the part of variance accounted for by the first dimension (corresponding to the dimension embedding the variability of generative models) increased from 51.5% ± 1.2% to 63.1% ± 1.0%, while the part of variance accounted for by the second dimension (which corresponds to the dimension hypothesized to bear the functional consequences of SC estimation errors) decreased from 12.2% ± 0.6% to 7.0% ± 0.4%.

Even though we excluded interhemispheric data from the SVD analysis, this approach was not fully satisfactory, since simulated FC was generated to begin with by computational models taking interhemispheric information from SC into account. Thus we might expect that intrahemispheric simulated FC could also suffer from SC estimation errors concerning interhemispheric and homotopic connections. In order to further tackle this issue in a heuristic manner, we performed a new set of simulations and analyses restricted to SC and cFC averaged across subjects, as we could not afford an analysis on individual data at this stage given the heavy computational burden of the simulations [68]. This precluded further bootstrap analysis and thus made the results only descriptive, but we had a directed hypothesis and were only looking for confirming the impact of failing to detect homotopic connections on the observed discrepancies between empirical and simulated FC. We manipulated the average SC by artificially setting homotopic connections to an arbitrary value (0.5). Two new batches of simulations across all seven computational models were run in order to generate simulated matrices of FC as predicted from the original average SC matrix on the one hand and, on the other hand, from the modified average SC matrix. We hypothesized that adding homotopic connections would increase the variance explained by the first linear dimension and decrease that of the second one in a standard SVD analysis. We observed that adding homotopic connections increased the part of variance explained by the first dimension from 67.2% to 78.4%, while it decreased the part of variance explained by the second dimension from 13.3% to 7.7% (Fig 5b).

Altogether this series of results supports the hypothesis that, independently from unmodeled differences that would be related to the complexity of the dynamics being captured, an incorrect estimation of SC, which is itself related to current limitation in DWI, plays an important role in the observed discrepancies between FC predicted by the different generative models and empirical FC. They also further support the hypothesis of a strong relationships between FC as measured and SC.

### Dynamic FC: Changing the time scale of the analyses nuances but does not change the overall picture

**Fig 6:**
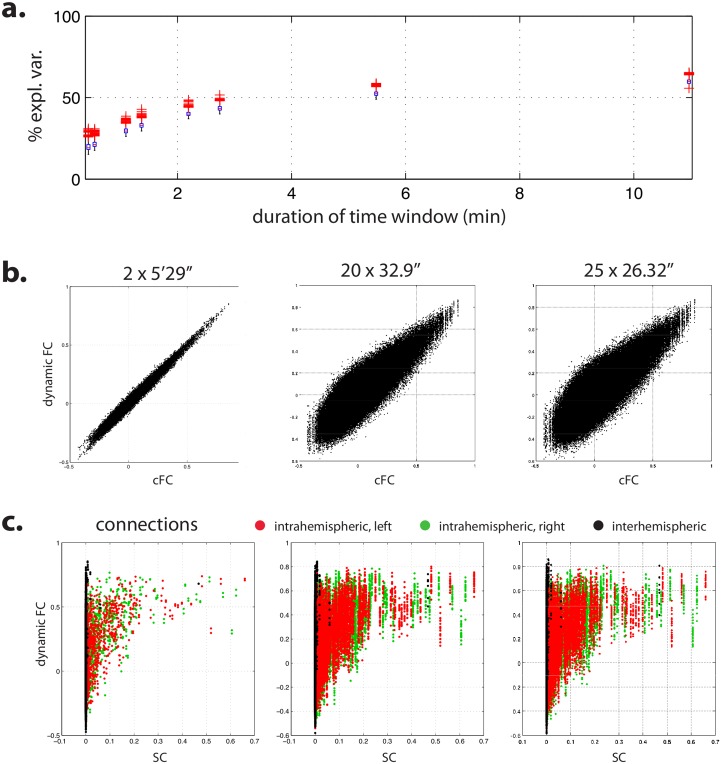
Dynamic FC. Bootstrap SVD extracted a reproducible linear space of dimension one for all window sizes but the shortest, where no reproducible linear space was found. **(a)** Part of variance explained by the first dimension as a function of the size of the time window used to compute dynamic FC. **(b)** First dimension of dynamic FC as a function of the first dimension of cFC. **(c)** First dimension of dynamic FC as a function of the first dimension of empirical SC.

In all the analyses so far, we computed FC over a whole rs-fMRI session, that is, over time series of about 11 minutes. We hypothesized that in resting state data acquired at arbitrary time points across different subjects, brain computation and dynamics would be maximally independent and that, consequently, such sampling strategy would foster the expression of putative differences in patterns related to this free state, and minimize the impact of more invariant constraints. However, it is possible that computing FC over time series of several minutes tends to bias the sensitivity of the analysis toward the most stationary aspects of the processes, thus missing the relevant variability because of the wrong choice of time scale. We thus conducted a bootstrap SVD analysis, concentrating on empirical data and using cFC, but this time on concatenated matrices of cFC independently computed over shorter time windows. Bootstrap SVD extracted a reproducible linear subspace with dimension 1 for all window sizes (from 32.9 s to 5 min 29 s) but the shortest (26.32 s), where no reproducible linear subspace could be extracted. The part of variance explained by the first dimension decreased however from 52.6% ± 1.6% for 2 windows of size 5 min 29 s to 21.5% ± 2.0% for 20 windows of size 32.9 s and 19.8% ± 2.4% for 25 windows of size 26.32 s (Fig 6a). This is to be compared to the 59.8% ± 1.6% of total variance explained by the analysis with cFC computed over the whole session. Dynamic FC featured a reproducible linear dimension that was similar to that of cFC, with a residual variance that increased as the window size decreased (Fig 6b and 6c).

### Inverting FC to infer SC: Proof of concept

One of the findings of these analyses is that FC from rs-fMRI, as measured with correlation, mutual information, or 3-way connectivity, contained a degenerate projection of the complexity and variability of the dynamics underlying brain computation, which reflects a robust and stable common core. This core of FC also appeared to be clearly related to SC, both considering empirical data and simulations from generative models in which the relation is explicit. We reasoned that the empirical relationship between SC and FC (as measures) suggests that their common features reflect the same underlying anatomical network. Given the customary issues of false negatives and false positives in standard DWI pipelines, measurements of FC from rs-fMRI might thus appear as a fair additional ground for (re)estimating SC in a more encompassing way. While developing and testing a valid and full-fledged procedure for SC estimation through inversion of FC is beyond the scope of this article, we here give a proof of concept in favor of the possibility of such a procedure.

The procedure relies on two main assumptions: (i) There is a simple, one-to-one relationship between SC and FC which is relatively insensitive to dynamical regimes (and that loads on the first component of the SVD); and (ii) deviation from this relationship (at the level of this first component) is mostly a consequence of incorrect estimation of SC. According to (i), one could quantify the relationship between SC and FC from simulations and then apply its inverse to empirical FC. More specifically, from the analyses, we were led to the conclusion that SVD on the pooled data extracted a first component that is a reflection of the underlying steady functional-anatomical organization, and a second one that is related to errors in SC estimation. We reasoned that, should DWI provide correct SC, the second component of SVD on the pooled data would be associated to vanishing variance, while the fraction of variance explained by the first component would increase to 1, and the relationship between SC and FC would appear on this component. As a consequence, the empirical data would mostly project on the first component. We then also postulated the relative stability of the relationship between SC and FC regardless of the quality of estimation of SC by DWI. In other words, a better estimation of SC would reduce the fraction of variance explained by the second component and increase the fraction of variance explained by the first component but not drastically change the relationship already observed between SC and FC on the first component.

**Fig 7:**
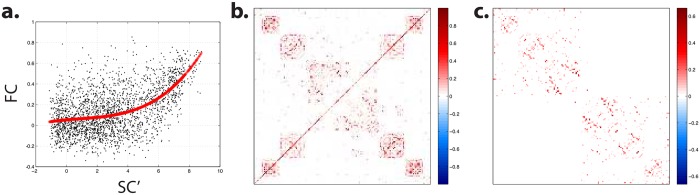
Inverting empirical FC. **(a)** Average projection of all (empirical and simulated) data onto first reproducible linear dimension as a function of the average projection of empirical SC onto its first reproducible linear dimension thresholded at SC ≥10^−6^ and transformed according to SC′ = ln(SC/0.0001) (dots). The red line stands for a 4-order polynomial interpolation approximation. **(b)** SC estimated by inverting the previous relationship applied to empirical cFC. **(c)** As a reference, average projection of empirical SC onto its first reproducible linear dimension.

Practically, we first quantified the relationship observed between the first reproducible linear dimension of empirical SC and that of the pooled data (Fig 7a), then applied the inverse of this transform to empirical FC (see Methods). Such an approach produced an estimated matrix of SC that was similar to, but richer than, the original DWI-based SC matrix, featuring more interhemispheric connections, especially homotopic ones (Fig 7b and 7c). Beyond this preliminary example, a Bayesian iterative scheme could be developed in the future, embedding a similar approach, and aiming at converging towards optimal estimates of SC.

## Discussion

As they stand FC metrics might thus only offer a degenerate measure of the brain’s complex dynamical interactions and be a limited entry point to study brain computation and its relation to cognition.

In summary, we found: 1) that simulated and empirical FC demonstrated a highly similar, invariant common core that could essentially be represented by one main linear direction which could explain about 58% of the total variance of empirical correlation based FC across subjects; 2) that this pattern of FC was rather well predicted by computational models taking SC as an input and was robust to the equations and fixed parameters of the underlying dynamics; 3) that measures of FC based on correlation, mutual information and 3-way interactions were quite similar regarding this linear subspace, and thus that the findings were not merely an artifact of the standard linear measure of ‘functional connectivity’; 4) that varying the time scale of the analyses did not eliminate this main linear direction; 5) and that this main dimension of FC was strongly related to SC, empirically and through generative rules, to the point that estimating SC from FC could be envisioned. The high level of degeneracy of measures of empirical FC was confirmed on a second independent dataset of 40 subjects from the HCP (http://www.humanconnectome.org/documentation/Q2/; see §2 of S1 Text).

Incidentally, we demonstrated that the observed relationship between correlation and mutual information in BOLD-related measures of FC overall behaved in agreement with the hypothesis of an underlying multivariate Gaussian process. Accordingly, the analytical relation $\text{mFC}=-\frac{1}{2}\ln\left(1-{\text{cFC}}^{2}\right)$ can be used as a rule of thumb in order to estimate mutual information from simple measures of correlation, though the residual variance remain substantial between the Gaussian model and the measures. 3-way interactions yielded similar findings and also suggested the existence of an underlying Gaussian process.

### Degeneracy of FC

From a theoretical standpoint, the existence of a low-dimensional approximation of FC from SVD implies that strong linear relationships control FC variability, whereas approximations of significantly higher dimension (or no approximation at all) would imply more complex relationships.

Our findings suggest that, to the least, FC as measured by these different metrics from rs-fMRI is a strongly degenerate representation of the potential complexity of underlying interactions. This relative degeneracy of FC was not simply attributable to the BOLD signal, whose SVD did not feature any main linear directions, but was a direct effect of measuring FC. Previous studies emphasized that a broad range of computational models generating time courses with quite different spectra and oscillatory properties featured limited yet similar overall predictive powers of simulated FC [54, 68]; such results also suggest that FC is degenerate with respect to dynamical regimes.

It remains that part of the variance was not reducible to this main linear dimension to an extent that depended on the metrics used to compute FC: according to SVD analyses, mutual information tended to explain less variance along the first linear dimension than correlation, and could also feature a second reproducible linear dimension. Likewise, even though the main linear dimension was still reproducibly present when reducing the time window of analysis for the computation of FC down to about 30 seconds, it explained less and less variance. It is difficult to assess how much this additional unexplained variance reflect increasingly worse performance in estimating FC or meaningful changes that reflect brain computation [31, 71].

### Relationship between SC and FC

Degrees of freedom between SC and FC could be expected, as adaptive brain computation implies dynamic interactions for the routing and integration of information. However, at the level of our measurement space, such degrees of freedom appeared reduced to a main linear dimension relating all observations. Subjects’ cognition may be free ranging in the resting state, but this did not manifest as irreducibly complex differences between independently generated patterns of FC. BOLD-related FC, in particular along 5–10 minute runs, appeared fundamentally dominated by SC, which itself appears dominated by one main linear dimension, explaining up to 86% of the variance across subjects. Altogether this emphasizes that anatomical connections of brain structures themselves must have a strong backbone that is invariant across subjects and that drives a relatively invariant net pattern of bidirectional information transfer. This pattern is reflected in FC and implies an overall bandwidth across communication channels that is a function of the quantity of fibers (empirically estimated by DWI-SC).

By manipulating SC, [54] demonstrated the importance of the integrity of SC and of the accuracy of its measurement on the predictive power of generative models for FC. Here, beyond predictive power, we further showed that the main differences between the patterns of simulated and empirical FC largely loaded on connections that were known to be poorly estimated in DWI tractography and that were absent in our SC matrices. Therefore the main factor limiting the predictive power of generative models might turn out to largely reduce to problems of estimation of SC (when we artificially added homotopic connections in our SC data, the variance explained by the first linear dimension increased from 67% to 78%). Improving tractography is therefore expected to greatly improve the fit of simulated FC to empirical FC [69, 70].

Now, in spite of their strong relationships, there are deeper systematic differences between SC and FC that need to be emphasized. FC as measured is the image of (linear or nonlinear) correlations among time-courses that are generated by an underlying dynamical process (either simulated or empirical). While being nominally channeled by SC, FC is ultimately induced by an underlying hidden weighting of SC, sometimes called ‘effective connectivity’ (EC) [72, 73]. This weighting of connections operates as multiplicative gains (which can be positive or negative) in the local transfer functions between connected regions, which themselves drive the overall dynamical process. Obviously, estimates of the specific weighting of EC cannot be simply inferred from DWI-based measurements of SC. However, based on the relationships demonstrated herein, procedures of inversion of FC, as envisioned and preliminarily explored above, could yield fair estimates of EC itself, which would be of great interest for neurocomputational modeling and inference.

Likewise, BOLD-related measures of FC could provide, after adequate inversion, the basis for novel multimodal approaches to tractography, supporting inference on existing fibers in the context of ambiguous diffusion signal based on EC or SC estimates from FC.

### Reproducibility, variability, SC, FC, and cognition

In the introduction, we referred to two bodies of literature, one showing the relative reproducibility of FC between subjects, and the other one showing that FC could be influenced by a wealth of factors, such as brain maturity, age, the global level of awareness, personality traits, the current mental state, recent experience, and time. In the present study, we found that the information contained in FC was rather degenerate and in a large part reflective of SC. Together with the fact that SC (as seen by DWI tractography) is rather reproducible across subjects, this is in agreement with studies showing the reproducibility of FC. As to the relative influence of the two main (anatomical and dynamical) factors on FC, our study hints that SC strongly dominated the signal and loaded on the first reproducible SVD component. Dynamical functional patterns (and noise) might load on the remaining components, which are not associated with reproducible patterns. The effect of some factors which cannot easily be associated with a modification of structural connections but are most likely related to a change in dynamical regime (e.g., the global level of awareness, the current mental state), are probably observed on the part that is not accounted for by the first reproducible SVD component. This distinction could be used to classify data accordingly, even though some factors might influence both structure and dynamics. A future study could apply the framework developed in the present manuscript to experimental settings where a variation of FC is observed and quantified concomitantly with a change in state (e.g., sleep or loss of consciousness [8, 74]), behavior, or cognition, and investigate how such change would affect the various singular values from the SVD and corresponding reproducible patterns of FC. In any case, an important point is that the range of variability available to FC is quite reduced compared to the same range at the level of neuronal processes or even at the level of the BOLD signal.

Among remaining questions for future studies, one may wonder whether the degeneracy of FC would be as strong with input signals offering more sensitivity to more complex and potentially faster dynamics than the slow and indirect BOLD signal, and how much it relates to the spatial scale of observation. While we showed that increasing the parcellation scale from 160 to 461 to 825 regions essentially led to similar conclusions in terms of degeneracy of FC, a more drastic change in scale together with a change in imaging method might provide complementary insight into the origin and function of brain interactions [75]. The same methodological framework could be applied to electrophysiological measurements and computational models directly, though measuring the required empirical data for the analysis at the scale of the matrix of SC could be challenging. We may also wonder if the brain’s ability to re-route information flow after brain damage, a process that might underlie mechanisms of functional recovery and be involved in resilience to neurological disorders, could not be related to a loosening of the relationship between SC and FC [5].

## Materials and Methods

In this section, we present a summary of the data used for the analysis. More information can be found in §1 of S1 Text or in [68].

### Data

#### Real data

Twenty one subjects were scanned for anatomical MRI, DWI, and resting-state fMRI. Data were preprocessed using a standard approach (see S1 Text, §1). The brain was parcellated using Freesurfer software (http://surfer.nmr.mgh.harvard.edu/) [76] and the procedure described in [77], with a parcellation of 160 regions. These regions were further subdivided into two sets of smaller compact regions, yielding two finer partitions of the cerebral cortex into 461 and 825 regions (about 6 and 3 cm^2^ each respectively). A probabilistic white matter fiber tracking method [78] implemented in FSL was used to track all possible connections between all pairs of regions. An index of structural connectivity between two regions was then defined as the proportion of fiber samples connecting these two regions per unit surface. This index was further divided by the average fiber length to reduce bias towards longer fibers. This structural connectivity index allowed to build an individual structural connectivity matrix ***D*** for each subject, *D*_*rs*_ being the structural connectivity index from regions *r* to region *s*, with no self-connections (i.e., *D*_*rr*_ = 0). ***D*** was then thresholded at 0.001; supra-threshold values were kept as such. In fMRI, the time series of all voxels within a given region were spatially averaged to form the representative signal of that region. Data were considered with and without regression of the global signal.

#### Simulations

We used 7 generative models: the SAR model, the Rate model, the Wilson-Cowan system, the Kuramoto model, the Fitzhugh-Nagumo model, the Neural-mass model, and the Spiking neuron model. All models took an SC matrix as input, and all but the SAR were taken as models of neuronal (rather than BOLD) activity. Simulated fMRI BOLD signal was obtained from simulated neuronal activity by means of the Balloon-Windkessel hemodynamic model [79, 80]. Global mean signal was then regressed out from each region’s time series. Finally, simulated FC was computed as Pearson correlation between simulated time series. For the SAR model, we directly computed simulated FC from the analytical expression of the covariance matrix. The global coupling parameter of each model was tuned so as to optimize predictive power on the average subject vis-a-vis empirical FC (see S1 Text, §1).

#### Measures of functional connectivity

Together with standard (i.e., correlation-based) FC, we considered three other measures in order to detect interactions that could potentially go undetected by correlation: mutual information (MI) for nonlinear interactions, 3-way connectivity (CT3) for interactions between three regions, and windowed correlation (or “dynamic FC”) for transient interactions.

Standard FC, which is optimal to capture linear bivariate relationships, was computed as the usual Pearson correlation between the BOLD time courses corresponding to any pair of regions, yielding 160 × 159/2 = 12720 coefficients for 160 regions, 461 × 460/2 = 106030 coefficients for 461 regions, and 825 × 824/2 = 339900 coefficients for 825 regions.

To investigate dynamic FC, we decomposed for each subject the signal into non-overlapping windows of identical size and computed FC on each window. All FCs corresponding to the same acquisition run were then concatenated. Window size varied in {8, 10, 20, 25, 40, 50, 100} sample points, corresponding to {25, 20, 10, 8, 5, 4, 2} time windows respectively of length between 26.32 s and 5 min 29 s and yielding 12720 coefficient per time window.

We also considered MI based functional connectivity. MI is a measure of interaction between two variables *X*_*i*_ and *X*_*j*_ that is defined as

$$I\left(X_{i},X_{j}\right)=\int p\left(x_{i},x_{j}\right)\;\ln\frac{p(x_{i},x_{j})}{p\left(x_{i}\right)\;p\left(x_{j}\right)}\;dx_{i}\;dx_{j}.$$

It is always positive, and is equal to 0 if and only if *X*_*i*_ and *X*_*j*_ are independent, that is,

$$p\left(x_{i},x_{j}\right)=p\left(x_{i}\right)\;p\left(x_{j}\right).$$

It is able to encompass a broader set of relationships than correlation, including nonlinear relationships. For a bivariate Gaussian distribution, MI can be expressed as

$$I\left(X_{i},X_{j}\right)=-\frac{1}{2}\ln\left(1-\rho_{ij}^{2}\right),$$

where *ρ*_*ij*_ is the correlation between *X*_*i*_ and *X*_*j*_. In this particular case, MI is an increasing function of the absolute value of *ρ*_*ij*_, with the specificity that a zero MI is equivalent to a zero correlation. MI is quite complex to estimate in the general case. We here relied on an estimator proposed by [81] and implemented in TIM 1.2.0 (http://www.cs.tut.fi/∼timhome/tim/tim.htm).

MI can be generalized to an *n*-way interaction measure between *n* variables, leading to a measure called either total correlation [82], multivariate constraint [83], multiple correlation [84], integration [26, 85], or multiinformation [86]. In particular, a 3-way connectivity measure can be computed for each triplet of variables as

$$I\left(X_{i},X_{j},X_{k}\right)=\int p\left(x_{i},x_{j},x_{k}\right)\;\ln\frac{p(x_{i},x_{j},x_{k})}{p\left(x_{i}\right)\;p\left(x_{j}\right)\;p\left(x_{k}\right)}\;dx_{i}\;dx_{j}\;dx_{k},$$

leading to a total of 160 × 159 × 158/6 = 669920 coefficients. CT3 can detect interaction patterns that bivariate measures such as correlations and MI could potentially overlook. For a trivariate Gaussian distribution, the expression of CT3 boils down to

$$\begin{array}{ccc} I(X_{i},X_{j},X_{k}) & = & -\frac{1}{2}\ln\left|\begin{array}{ccc} 1 & \rho_{ij} & \rho_{ik}\\ \rho_{ij} & 1 & \rho_{jk}\\ \rho_{ik} & \rho_{jk} & 1 \end{array}\right|\\ & = & -\frac{1}{2}\ln\left[1+2\rho_{ij}\rho_{jk}\rho_{ik}-\left(\rho_{ij}^{2}+\rho_{jk}^{2}+\rho_{ik}^{2}\right)\right]. \end{array}$$ (2)

#### Datasets

We relied on 10 distinct datasets (see Table 1):

- Dataset #1: empirical data.
- Dataset #2: empirical data with no regression of the global signal.
- Dataset #3: empirical data with 461 regions.
- Dataset #4: empirical data with 825 regions.
- Dataset #5: empirical data with permutation of all 12720 links (5a), all 160 regions (5b), or all 80 pairs of homotopic regions (5c).
- Dataset #6: simulated data. For each subject, the data consisted of 7 vectors of identical size, one for each generative model.
- Dataset #7: all (i.e., empirical and simulated) data for each connectivity measure.
- Dataset #8: we only considered the 6320 intrahemispheric connections of FC.
- Dataset #9: this analysis required computation of the residual FC patterns, obtained as the difference between each empirical FC and any simulated FC, resulting in 147 residual FC patterns of dimension 12720.
- Dataset #10: we considered data from the average subject, that is: (i) simulated data generated by applying each of the 7 generative models to the SC obtained by averaging individual SC matrices over subjects, and (ii) FC obtained by averaging individual empirical FC matrices over subjects. In one setting, the average CS was manipulated by artificially setting homotopic connections to a given value (0.5) in the connectivity matrix.
- Dataset #11: analysis of dynamic FC on the empirical data.

**Table 1:** Data. Summary of data used for the various analyses. GSR: global signal regression.

Dataset	Type of data			GSR	# regions	Connections	Measure	Dimension
#1	empirical	individual	whole session	yes	160	all	SC correlation MI CT3 BOLD	12720 × 21 12720 × 21 12720 × 21 669920 × 21 32000 × 21
#2	empirical	individual	whole session	no	160	all	correlation	12720 × 21
#3	empirical	individual	whole session	yes	461	all	correlation	106030 × 21
#4	empirical	individual	whole session	yes	825	all	correlation	339900 × 21
#5a #5b #5c	empirical empirical empirical	individual individual individual	whole session whole session whole session	yes yes yes	160 160 160	all (links permuted) all (links permuted) all (links permuted)	correlation correlation correlation	12720 × 21 12720 × 21 12720 × 21
#6	simulated	individual	whole session	yes	160	all	correlation MI CT3	12720 × 147 12720 × 147 669920 × 147
#7	all	individual	whole session	yes	160	all	correlation MI	12720 × 168 12720 × 168
#8	all	individual	whole session	yes	160	intrahemispheric	correlation	6320 × 168
#9	residual	individual	whole session	yes	160	all	correlation	12720 × 147
#10	all	average subject	whole session	yes	160	all, manipulated	correlation	12720 × 8
#11	empirical	individual	2 × 5’29” 4 × 2’44.5” 5 × 2’11.6” 8 × 1‘22.25’ 10 × 1’5.8” 20 × 32.9’ 25 × 26.32”	yes	160	all	correlation	25 440 × 21 50 880 × 21 63 600 × 21 101 760 × 21 127 200 × 21 254 400 × 21 318 000 × 21

### SVD analyses

All datasets previously mentioned were analyzed in a similar fashion using singular value decomposition (SVD). For all but Dataset #9, we applied bootstrap SVD.

#### Standard SVD

SVD is a technique that extracts the main (linear) directions of variability of a data set. In SVD, a given *n*-by-*m* (*n* > *m*) data matrix ***X*** is decomposed into [87]

$$X=USV^{t},$$

where ***U*** and ***V*** are *n*-by-*n* and *m*-by-*m* orthogonal matrices, respectively, and ***S*** is an *n*-by-*m* rectangular diagonal matrix with nonnegative diagonal elements (*s*_1_, …, *s*_*m*_), *s*_*i*_ > *s*_*i*+1_, called singular values. The columns ***u***_*i*_ of ***U*** are called the left-singular vectors. They form an orthonormal basis where the projection of the data on each ***u***_*i*_ can account for a fraction of the variance that is equal to $s_{i}^{2}/\sum_{j=1}^{m}s_{j}^{2}$. Since the singular values are ranked in decreasing order, the ***u***_*i*_’s are associated with decreasing fractions of variance. Furthermore, the projection of ***X*** onto the *d* first left-singular vectors (***u***_1_, …, ***u***_*d*_) has two key features: it is the best *d*-dimensional approximation of ***X*** (in a least square sense) and, equivalently, the *d*-dimensional approximation of ***X*** which explains the most of its variance [87].

#### Bootstrap SVD

To determine the reproducibility of SVD analysis, we applied a strategy based on bootstrap SVD [88]. We performed *B* = 1000 bootstrap replicates in which we bootstrapped the 21 subjects, and each bootstrap replicate was decomposed using standard SVD. We then constructed the bootstrap confidence interval of each *s*_*i*_ from the bootstrap values $\left(s_{i}^{\left[1\right]},\ldots ,s_{i}^{\left[B\right]}\right)$. Each *s*_*i*_ whose bootstrap confidence interval did not overlap with that of *s*_*i*+1_ could be reproducibly associated with a reproducible linear subspace with dimension one spanned by the corresponding left-singular vector ***u***_*i*_. Denoting by *s*_*d*_ is the last such singular value, then a fraction $\left(\sum_{i=1}^{d}s_{i}^{2}\right)/\left(\sum_{i=1}^{m}s_{i}^{2}\right)$ of the variance could be reproducibly explained by a linear subspace spanned by the *d* first left-singular vectors (***u***_1_, …, ***u***_*d*_). This linear subspace had the key feature of being the linear subspace of dimension *d* explaining the most variance or, equivalently, best explaining the data in a least square sense.

#### Characterization of reproducible linear dimensions

Once the number of reproducible linear dimensions was extracted using bootstrap SVD, we characterized each dimension *i* by the corresponding left-singular vector ***u***_*i*_, transformed back into a matrix and weighted by a certain proportionality factor. In the general case, this proportionality factor was computed as the average projection of the data onto ***u***_*i*_ or, equivalently, the projection onto ***u***_*i*_ of the average data (since averaging and projection are linear operators, both operators can be exchanged)

$$\frac{1}{m}\sum_{j=1}^{m}u_{i}^{t}x_{j}=u_{i}^{t}\left(\frac{1}{m}\sum_{j=1}^{m}x_{j}\right),$$

where the ***x***_*j*_’s are the columns of ***X***. In the specific case where the data consisted of all (i.e., empirical and simulated) FC patterns, the representative FC pattern along the second singular vector was taken as the average projection of the empirical data only, in order to provide a better contrast between large and low values of ***u***_*i*_.

### Inferring SC from empirical FC

In order to infer SC from empirical FC, we first quantified the relationship observed between the average projection of empirical SC onto its first left-singular vector and the average projection of all FC onto its first left-singular vector. Empirical observations suggested to consider only values of SC ≥10^−6^ and to apply the following transformation:

$${\text{SC}}^{\prime }=\ln\left(\frac{\text{SC}}{0.0001}\right).$$

We then approximated the relation between SC’ and FC through 4-order polynomial interpolation, FC ≈ *P*_4_(SC′). Estimation of SC through inversion of the relationship between SC and FC was then carried out using the relationship

$$\hat{\text{SC}}=0.001\;\exp\left[P_{4}^{-1}\left(\text{FC}\right)\right].$$

## Supporting Information

S1 Text (PDF)
