# Supplementary material for: Functional Connectivity’s Degenerate View of Brain Computation
Source: PLoS Comput Biol. 2016 Oct 13;12(10):e1005031. doi: 10.1371/journal.pcbi.1005031 (PMC5063374; doi:10.1371/journal.pcbi.1005031)
Supplement: S1 Text — (PDF) [file pcbi.1005031.s001.pdf]

Supplementary Information

1 Materials and methods

1.1 Real data

Acquisition. Twenty one right-handed healthy volunteers were recruited within local community (11 males, mean age 22 ± 2.4 years). All participants gave written informed consent and the protocol was approved by the local ethics committee. Data were acquired using a 3 T Siemens Trio TIM MRI scanner (CENIR, Paris, France). Resting-state fMRI series were recorded using a single-shot, gradient-recalled echo-planar imaging sequence (repetition time TR: 3290 ms; echo time TE: 31 ms; field of view: 192 × 192 × 115 mm; matrix size: 128 × 128 × 46; flip angle: 90°; $1.5 \times 1.5 \times 2.5$ mm^3 voxels; 46 contiguous slices). Two hundred fMRI volumes were acquired, during 11 min. The subjects were instructed to remain eyes closed and to reduce any mental effort. DWI data were recorded using a single-shot, echo planar imaging sequence (TR: 13 s; TE: 121 ms; 2 mm^3 isotropic voxels; 68 contiguous slices). Fifty encoding directions with $b = 1000$ s/mm^2 and a non-weighted image were acquired for each subject. A three-dimensional, T_1-weighted, magnetization prepared rapid gradient-echo volume was also acquired during the same scanning session (TR: 2.3 ms; TE: 2.98 ms; 1.1 mm^3 isotropic voxels). fMRI data were preprocessed using SPM5 software[1]. For each subject, the first 4 fMRI volumes were discarded to allow for T_1 equilibration, and the remaining 196 fMRI volumes were corrected for slice-timing and head motion, excessive motion (greater than 3 mm or 3°) was not present in any of the subjects' scans. The resulting data were then spatially smoothed using an isotropic 6 mm full-width-at-half-maximum Gaussian kernel. DWI images were corrected for eddy-current distorsions using FSL, release 4.1[2] (Smith et al., 2004). Spatial normalization using linear transformations (combination of 3 translations, 3 rotations and 1 scale factor), between fMRI and DWI data and the anatomical volume, were computed for each subjects using FSL.

[1] http://www.fil.ion.ucl.ac.uk/spm/software/spm5/
[2] http://www.fmrib.ox.ac.uk/fsl/

Regions of interest. The T_1-weighted anatomical volume of each subject was parcellated using Freesurfer[3] (Fischl et al., 2004) and the procedure described in Hagmann et al. (2008). The procedure segmented the brain into gray matter and white matter compartments, and distinguished cortical and sub-cortical structures. A labeled cortical surface from an average template brain was registered onto the individual cortical surfaces, yielding a partition of the cerebral cortex of each subject into 160 regions (80 per hemisphere). This regional partition was registered using linear transformations previously calculated to the DWI and rsfMRI data of the subjects in order to derive corresponding matrices of SC and FC respectively.

Anatomical wiring. To quantify SC, we used the probabilistic white matter fiber tracking method (Behrens et al., 2007) implemented in FSL to track all possible connections between all pairs of regions. For every voxel of the white matter we initiated 500 fiber samples. Starting points were chosen randomly within the voxel space. Initial fiber orientation was randomly chosen and then fiber grew in the two opposite directions with a propagation step set at 0.5 mm and a maximal fiber curvature at 80° (no anisotropy constraint). Fiber tracking was stopped when samples reached the cortical surface. An index of structural connectivity between two regions was then defined as the proportion of fiber samples connecting these two regions per unit surface. This index was further divided by the average fiber length to account for the bias of the method towards longer fibers. This structural connectivity index allowed us to build a structural connectivity matrix $\boldsymbol{D} = (D_{ij})$ for each subject, D_{ij} being the structural connectivity index from region i to region j, with no self-connections (i.e. $D_{ii} = 0$). $\boldsymbol{D}$ was then thresholded at 0.001; supra-threshold values were conserved as such. Likewise, we built a matrix $\boldsymbol{L} = (L_{ij})$ for each subject, with entry L_{ij} corresponding to the average fiber length between regions i and j.

BOLD signal. The time series of all voxels within a given region were spatially averaged to form the representative BOLD signal of that region. To remove spurious sources of variance, linear and quadratic drifts, motion parameters, averaged ventricular, white matter and global brain signals were regressed out, and finally time series were low-pass filtered (<0.1 Hz) (Fox et al., 2009; Van Dijk et al., 2010).

1.2 Simulations

Models. We used seven generative models possessing various levels of complexity: the SAR model, a purely spatial model with no dynamics that expresses BOLD fluctuations within one region as a linear combination

[3]http://surfer.nmr.mgh.harvard.edu/

of the fluctuations in other regions; the Wilson–Cowan system, a popular model integrating excitatory and inhibitory neuronal populations; a rate model, which is a simplified version of the Wilson–Cowan system obtained by considering exclusively the excitatory population; the Kuramoto model, which simulates coupled oscillators; the Fitzhugh–Nagumo model, a reduction of the Hodgkin and Huxley model aiming at reproducing complex behaviors with explicit conductance-based dynamics; the neural-mass model, also based on models of conductances and featuring strong biophysiological constraints; and finally, the model of spiking neurons, the most constrained model in the current study, which models neuron populations as attractors. For most models, all parameters were taken from the original papers, except the global coupling strength which was optimized separately for each model (see below). For more details on the models, see Messé et al. (2015b).

From SC to BOLD via simulated neuronal activity. All models took a SC matrix as input, and all but the SAR model were explicit models of neuronal activity over time. Simulated fMRI BOLD signals were obtained from simulated neuronal activity by means of the Balloon–Windkessel hemodynamic model (Larter et al., 1999; Friston et al., 2003). Global mean signal was then regressed out from each region's time series. Finally, simulated FC was computed as the pairwise Pearson correlation between simulated time series. For the SAR model, simulated FC could be directly derived from the analytical expression of the covariance matrix (Messé et al., 2014, 2015a,b).

Numerical details of simulations. All simulations were performed in Matlab (The MathWorks Inc., Natick, MA), except for the spike model that had been implemented in the C language. The SAR model provides a closed form for the covariance matrix (Messé et al., 2014, 2015a,b) that can be used to directly compute the predicted functional connectivity. Dynamical models were simulated at a sampling frequency of 10 kHz. Simulations of the rate, Wilson–Cowan, Kuramoto, and Fitzhugh–Nagumo models relied on the Euler integration scheme, while Matlab ordinary differential equation solver was used for the neural-mass model. The resulting data were then downsampled to a sampling frequency of 1 kHz. The data corresponding to the first 20 s of the simulations were discarded from the analysis to avoid initial transient dynamics, resulting in 8 min of simulated brain activity. Simulated fMRI BOLD signal was obtained from neuronal activity by means of the Balloon–Windkessel hemodynamic model with a final sampling frequency of 2 Hz (Friston, 2003).

Parameter optimization. All models took a normalized form of the SC matrix as an input, as well as the value of a global coupling strength parameter over all pairs of regions. We performed a model-specific optimization

step over this latter parameter. For each model independently, we generated data with different matrix normalization strategies and values for the coupling parameter and saved the configuration that maximized predictive power. For normalization of SC, we considered 2 approaches: spectral and row normalizations (Barnett et al., 2009). Spectral normalization consists in dividing the SC matrix by its spectral radius, i.e., the largest absolute value of its eigenvalues. Row normalization imposes that the matrix rows sum to 1 (Tononi et al., 1994). For the coupling parameter, we used known bounds whenever it was documented (for the SAR and Rate models) and had a purely exploratory approach otherwise. Optimization was performed separately for each model on the average subject to limit computational burden. For all the remaining parameters, specific for each model, values were taken mostly from the original papers.

2 Supplementary analysis

2.1 Data

Data from 40 healthy, unrelated adults were obtained from the second quarter release (Q2, June 2013) of the Human Connectome Project (HCP) database[4]. The multimodal MRI data consisted of structural MRI, resting-state functional MRI (rs-fMRI), and diffusion MRI (dMRI), collected on a 3 T Skyra scanner (Siemens, Erlangen, Germany) using a 32-channel head coil. All scanning parameters are detailed and motivated in Van Essen et al. (2013). Multimodal MRI data were downloaded in a preprocessed form, that is, after the images had undergone the minimal preprocessing pipeline (v2). The details of this pipeline have been described previously (Fischl, 2012; Jenkinson et al., 2012; Glasser et al., 2013).

We used the same atlas as for the original data to parcellate brain into areas (160 regions). The preprocessed data were then fed into the same pipeline to extract both SC (see anatomical wiring section) and FC (see BOLD signal section).

2.2 Results

Bootstrap SVD extracted one reproducible linear space for SC, cFC and mFC. That space accounted for 90.9% ± 0.3% (for SC), 61.8% ± 1.1% (for cFC) and 79.6% ± 0.5% (for mFC) of the total variance.

[4]http://www.humanconnectome.org/documentation/Q2/

3 Views on anatomy

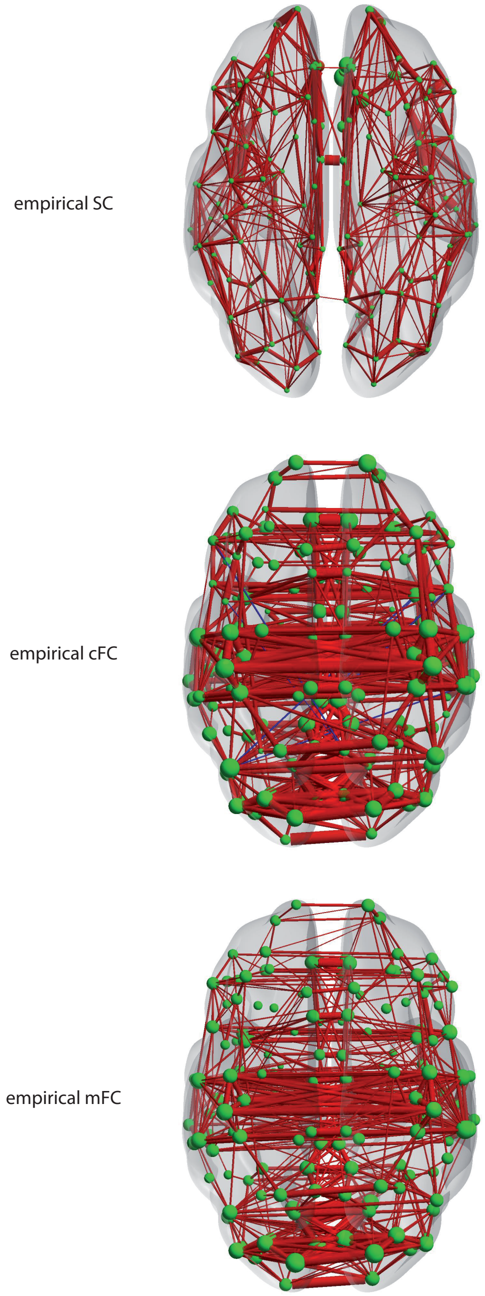

Figure 1: **Reproducible dimensions projected onto anatomical template for bootstrap SVD on empirical data.**

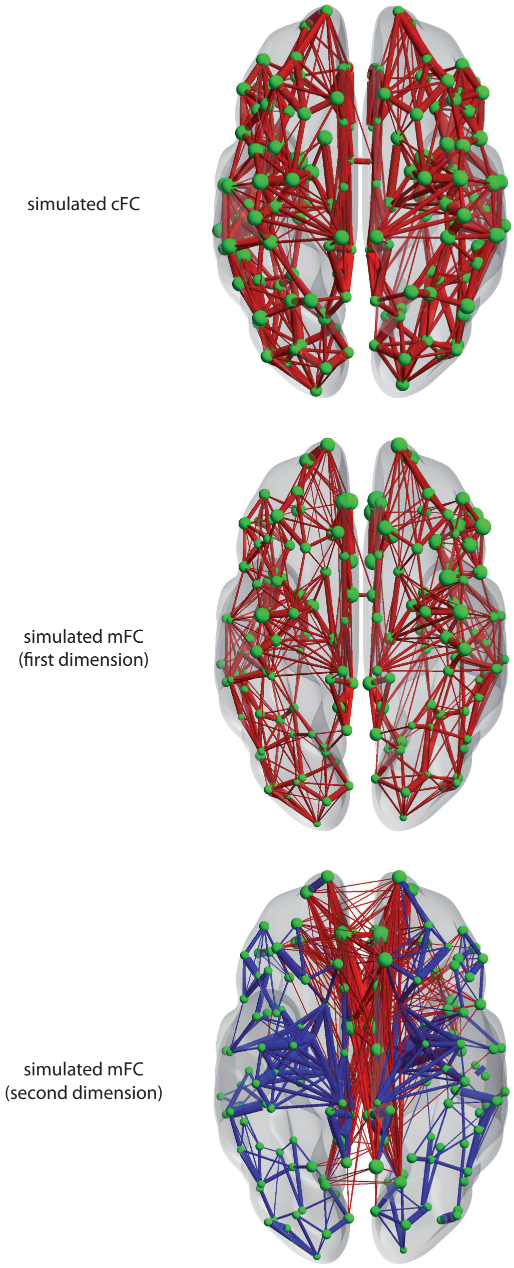

Figure 2: **Reproducible dimensions projected onto anatomical template for bootstrap SVD on simulated data.**

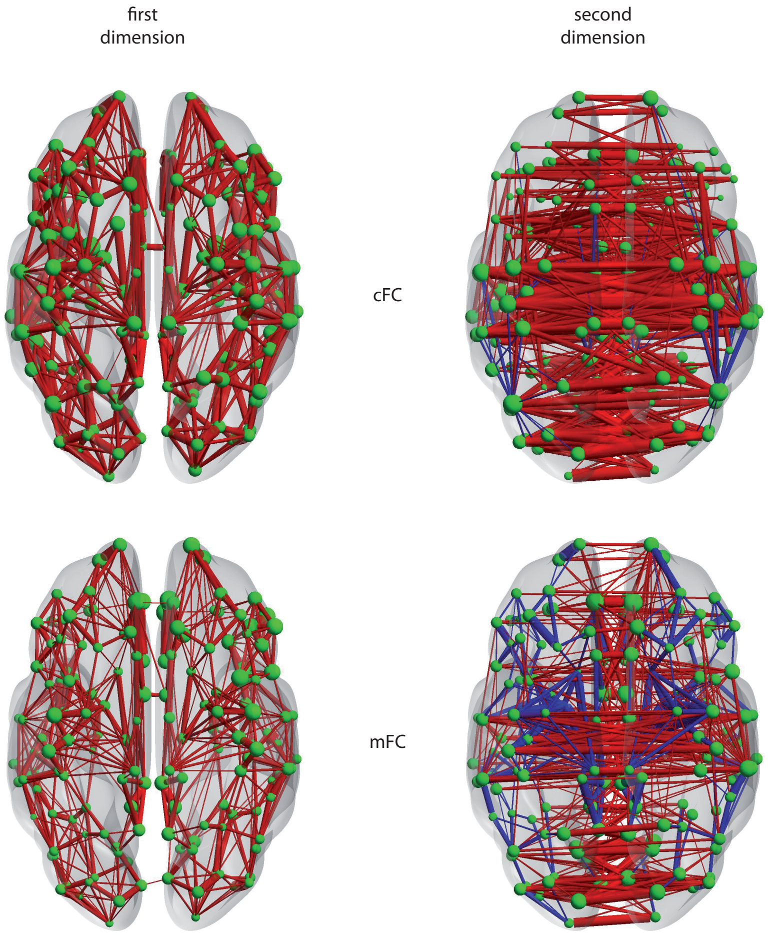

Figure 3: **Reproducible dimensions projected onto anatomical template for bootstrap SVD on pooled (empirical and simulated) data.**

References

Barnett, L., Buckley, C. L., Bullock, S., 2009. Neural complexity and structural connectivity. Physical Review E 79 (5), 051914.

Behrens, T. E. J., Berg, H. J., Jbabdi, S., Ruschworth, M. F. S., Woolrich, M. W., 2007. Probabilistic diffusion tractography with multiple fibre orientation: What can we gain? NeuroImage 34 (1), 144–155.

Fischl, B., 2012. FreeSurfer. NeuroImage 62 (2), 774–781.

Fischl, B., van der Kouwe, A., Destrieux, C., Halgren, E., Ségonne, F., Salat, D. H., Busa, E., Seidman, L. J., Goldstein, J., Kennedy, D., Caviness, V., Makris, N., Rosen, B., Dale, A. M., 2004. Automatically parcellating the human cerebral cortex. Cerebral Cortex 14 (1), 11–22.

Fox, M. D., Zhang, D., Snyder, A. Z., Raichle, M. E., 2009. The global signal and observed anticorrelated resting state brain networks. Journal of Neurophysiology 101, 327–383.

Friston, K. J., 2003. Learning and inference in the brain. Nature Neuroscience 16 (9), 1325–1352.

Friston, K. J., Harrison, L., Penny, W., 2003. Dynamic causal modelling. NeuroImage 19 (4), 1273–1302.

Glasser, M. F., Sotiropoulos, S. N., Wilson, J. A., Coalson, T. S., Fischl, B., Andersson, J. L., Xu, J., Jbabdi, S., Webster, M., Polimeni, J. R., Van Essen, D. C., Jenkinson, M., 2013. The minimal preprocessing pipelines for the human connectome project. NeuroImage 80, 105–124.

Hagmann, P., Cammoun, L., Gigandet, X., Meuli, R., Honey, C. J., Wedeen, V. J., Sporn, O., 2008. Mapping the structural core of human cerebral cortex. PLoS Biology 6, e159.

Jenkinson, M., Beckmann, C. F., Behrens, T. E., Woolrich, M. W., Smith, S. M., 2012. FSL. NeuroImage 62 (2), 782–790.

Larter, R., Speelman, B., Worth, R. M., 1999. A coupled ordinary differential equation lattice model for the simulation of epileptic seizures. Chaos 9, 795–804.

Messé, A., Benali, H., Marrelec, G., 2015a. Relating structural and functional connectivity in MRI: A simple model for a complex brain. IEEE Transactions on Medical Imaging 34 (1), 27–37.

Messé, A., Rudrauf, D., Benali, H., Marrelec, G., 2014. Relating structure and function in the human brain: relative contributions of anatomy, stationary dynamics, and non-stationarities. PLoS Computational Biology 10 (3), e1003530.

Messé, A., Rudrauf, D., Giron, A., Marrelec, G., 2015b. Predicting functional connectivity from structural connectivity via computational models using MRI: an extensive comparison study. NeuroImage 111, 65–75.

Smith, S. M., Jenkinson, M., Woolrich, M. W., Beckmann, C. F., Behrens, T. E. J., Johansen-Berg, H., Bannister, P. R., De Luca, M., Drobnjak, I., Flitney, D. E., Niazy, R. K., Saunders, J., Vickers, J., Zhang, Y., De Stefano, N., Brady, J. M., Matthews, P. M., 2004. Advances in functional and structural MR image analysis and implementation as FSL. NeuroImage 23 (Supplement 1), S208–S219.

Tononi, G., Sporns, O., Edelman, G. M., 1994. A measure for brain complexity: relating functional segregation and integration in the nervous system. Proceedings of the National Academy of Sciences of the U.S.A. 91 (11), 5033–5037.

Van Dijk, K. R. A., Hedden, T., Venkataraman, A., Evans, K. C., Lazar, S. W., Buckner, R. L., 2010. Intrinsic functional connectivity as a tool for human connectomics: theory, properties, and optimization. Journal of Neurophysiology 103, 297–321.

Van Essen, D. C., Smith, S. M., Barch, D. M., Behrens, T. E. J., Yacoub, E., Ugurbil, K., 2013. The WU-Minn human connectome project: an overview. NeuroImage 80, 62–79.
